# CellexalVR: A virtual reality platform to visualize and analyze single-cell omics data

**DOI:** 10.1016/j.isci.2021.103251

**Published:** 2021-10-27

**Authors:** Oscar Legetth, Johan Rodhe, Stefan Lang, Parashar Dhapola, Mattias Wallergård, Shamit Soneji

**Affiliations:** 1 Division of Molecular Hematology, BMC, Lund University, 22690 Lund, Sweden; 2 Lund Stem Cell Center, Lund University, 22184 Lund, Sweden; 3 Department of Design Sciences, LTH, Lund University, 22100 Lund, Sweden

**Keywords:** Bioinformatics, Computer science, Omics, Software engineering, Systems biology, Transcriptomics

## Abstract

Single-cell RNAseq is a routinely used method to explore heterogeneity within cell populations. Data from these experiments are often visualized using dimension reduction methods such as UMAP and tSNE, where each cell is projected in two or three dimensional space. Three-dimensional projections can be more informative for larger and complex datasets because they are less prone to merging and flattening similar cell-types/clusters together. However, visualizing and cross-comparing 3D projections using current software on conventional flat-screen displays is far from optimal as they are still essentially 2D, and lack meaningful interaction between the user and the data. Here we present CellexalVR (www.cellexalvr.med.lu.se), a feature-rich, fully interactive virtual reality environment for the visualization and analysis of single-cell experiments that allows researchers to intuitively and collaboratively gain an understanding of their data.

## Highlights

• Single-cell experiments are often visualized when embedded into three dimensions
• CellexalVR is a virtual reality environment to visualize all data simultaneously
• Teams can analyze single-cell experiments together in VR regardless of location

## Graphical abstract

**Figure:**
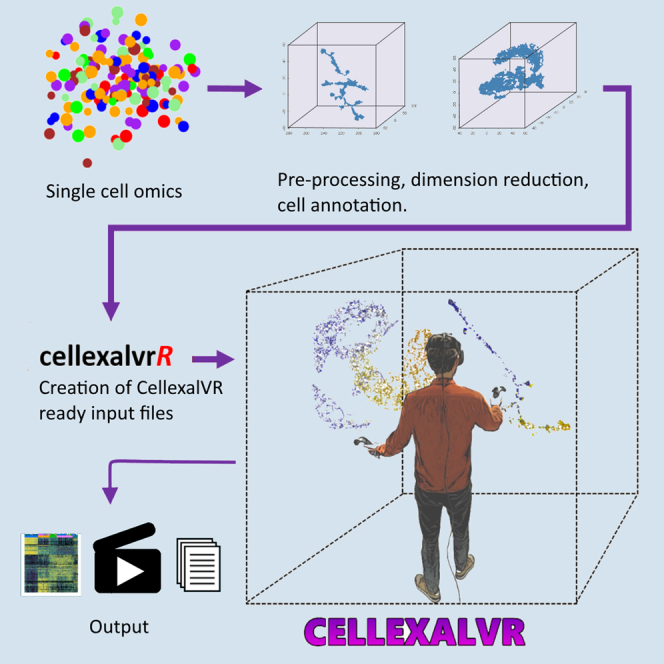

## Introduction

The analysis of single-cell RNAseq data (scRNAseq) is often performed using scripting, primarily using packages for the R/Python languages such as monocle (Trapnell et al., 2014), Seurat (Stuart et al., 2019) and SCANPY (Wolf et al., 2018) among others. A common step in the process is dimension reduction (DR) where cells are positioned in two/three dimensional space to visualize heterogeneity within the assayed populations. Many methods are available to do this (e.g, tSNE (Van Der Maaten and Hinton, 2008) and UMAP (Becht et al., 2019)), therefore several methods can be implemented during the course of an analysis and compared. Furthermore, each method has user-definable hyperparameters so outputs from the same methods also require comparison. Cells projected onto three dimensions can afford greater visual power to resolve the spatial arrangement of the cells and the clusters they form (Qadir et al., 2020), particularly when cell-types are similar but distinct.

Many tools are available to visualize scRNAseq data (Cakir et al., 2020), and published single-cell datasets are often accompanied by a webtool where 3D DR plots can be explored in a rudimentary fashion (Packer et al., 2019; Nestorowa et al., 2016) highlighting their utility. The visualization of 3D projected data is currently restricted to conventional computer monitors which have several shortcomings. The plots often have limited viewing angles because of them having only a single point of rotation at the center, making it difficult to view populations on the periphery. Importantly, only one projection can be loaded in the same window meaning direct comparisons between several 3D reductions cannot be made easily. Another drawback is these 3D plots are often not interactive, so for example, selecting cells for further analysis is not possible.

To solve these issues, VR is emerging as a potent tool to visualize 3D scientific data. Researchers see their data as true 3D objects, and with their hands in the same virtual space they have unlimited interaction possibilities. Examples of the application of VR include single-molecule localization microscopy (Blanc et al., 2020; Spark et al., 2020), neuron mapping (Wang et al., 2019), confocal microscopy (Theart et al., 2017; Stefani et al., 2018), molecular structures (Xu et al., 2020; Doutreligne et al., 2014), and network visualization (Pirch et al., 2021).

## Results

**Figure 1:**
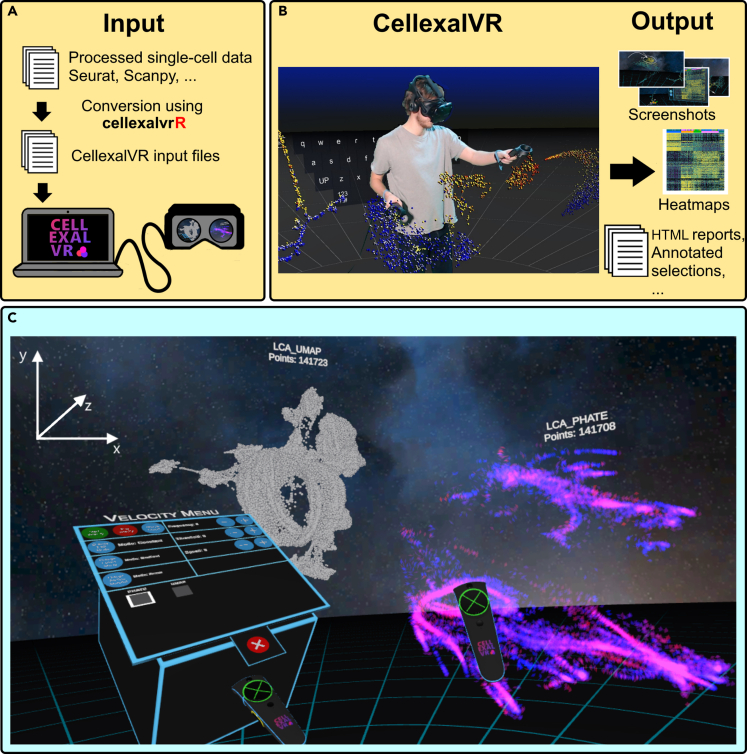
An overview of CellexalVR (A) Single-cell data is processed using a tool of the users choice (for example Seurat/Scanpy) and these are then converted to CellexalVR input files using our cellexalvrR R package. (B) A mixed-reality shot of CellexalVR showing the user in relation to the data in VR from which objects such as heatmaps and custom cell annotations can be exported. After a session has finished, an HTML report of actions performed in CellexalVR is automatically generated. (C) An example scene from the users’ point of view in CellexalVR. The hand controllers are seen in foreground, the left one showing the menu system. A UMAP and PHATE reduction of the Liver Cell Atlas is shown where our animated RNA velocity visualization has been activated on the PHATE mapping (Video S4).

Here we present CellexalVR, a free and open-source virtual reality platform for the visualization and analysis of single-cell data. By placing all DR plots and associated metadata in VR we have created an immersive, feature-rich, and collaborative environment to explore and analyze scRNAseq experiments. Many of these features have been suggested and refined by the single-cell community to optimize their ease-of-use and maximize utility. Single-cell data is processed using any method preferred by the user (Seurat/Scanpy for example) after which the resulting object is converted to a set of CellexalVR input files using our accompanying R package called cellexalvrR (Figure 1A). Although cellexalvrR is agnostic with respect to the processing method used, we have provided simple functions for the conversion of Seurat and Scanpy objects given their current popularity. In CellexalVR, the user coexists with the DR plots in virtual space where they can be handled as physical objects (Figure 1B). This is done using in-game hand controllers which also harbor the menu system where the features of CellexalVR can be activated (Figure 1C). Multiple DR plots can be visualized simultaneously and cross-compared with ease, for example, cells of interest in a tSNE plot can be captured with a hand gesture and traced to their counterparts in a UMAP allowing the user to directly determine the differences between reduction methods.

**Figure 2:**
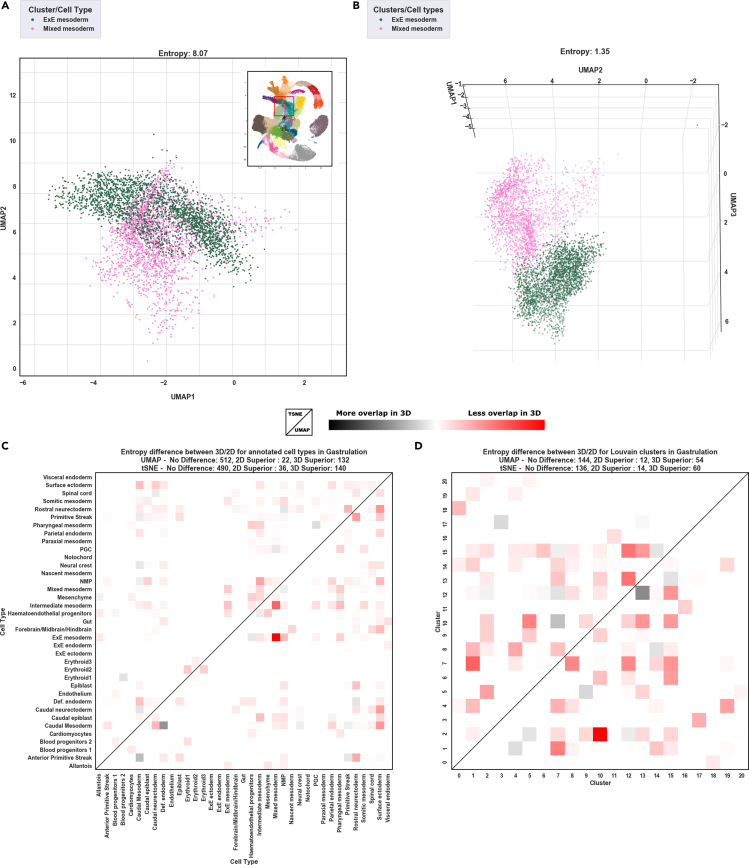
The benefits of 3D projections for complex single-cell data (A and B)(A) Two mesoderm types during mouse organogenesis overlap in a UMAP when projected onto two dimensions, where, in (B) they are transcriptionally distinct when projected onto three. (C) shows a pairwise comparison of cell type overlap using tSNE (upper diagonal) and UMAP (lower diagonal). Color shows the difference in entropy where red signifies lower cell type collision/entropy in the 3D projection and gray denotes lower cell type collision in the 2D projection, where it can be seen projection onto 3D overall has greater resolving power over 2D. (D) Shows the same pairwise test of overlap using a Louvain clustering of the cells.

As datasets become progressively larger and more complex, embedding the data over three-dimensions can have a greater visual advantage as it minimizes collapsing of similar cell-types/clusters allowing any separation to be seen clearly. To demonstrate this Figure 2A shows two subtypes of mesoderm during mouse gastrulation (Pijuan-Sala et al., 2019) that overlap in a 2D UMAP, however, the same data projected over three-dimensions shows them to be distinct (Figure 2B). We quantified this by systematically comparing pairwise the overlap between cell-types and clusters using entropy as a measure of mixing, whereas for cell-types and clusters (Figures 2C and 2D respectively) reduction to three-dimensions resulted in less overlap than in two-dimensions. This observation was held while using both UMAP (lower half) and tSNE (upper). We saw this trend consistently when applying this procedure to two further large datasets where we also used convex hulls as a second measure of overlap (Figures S1 and S2).

**Figure 3:**
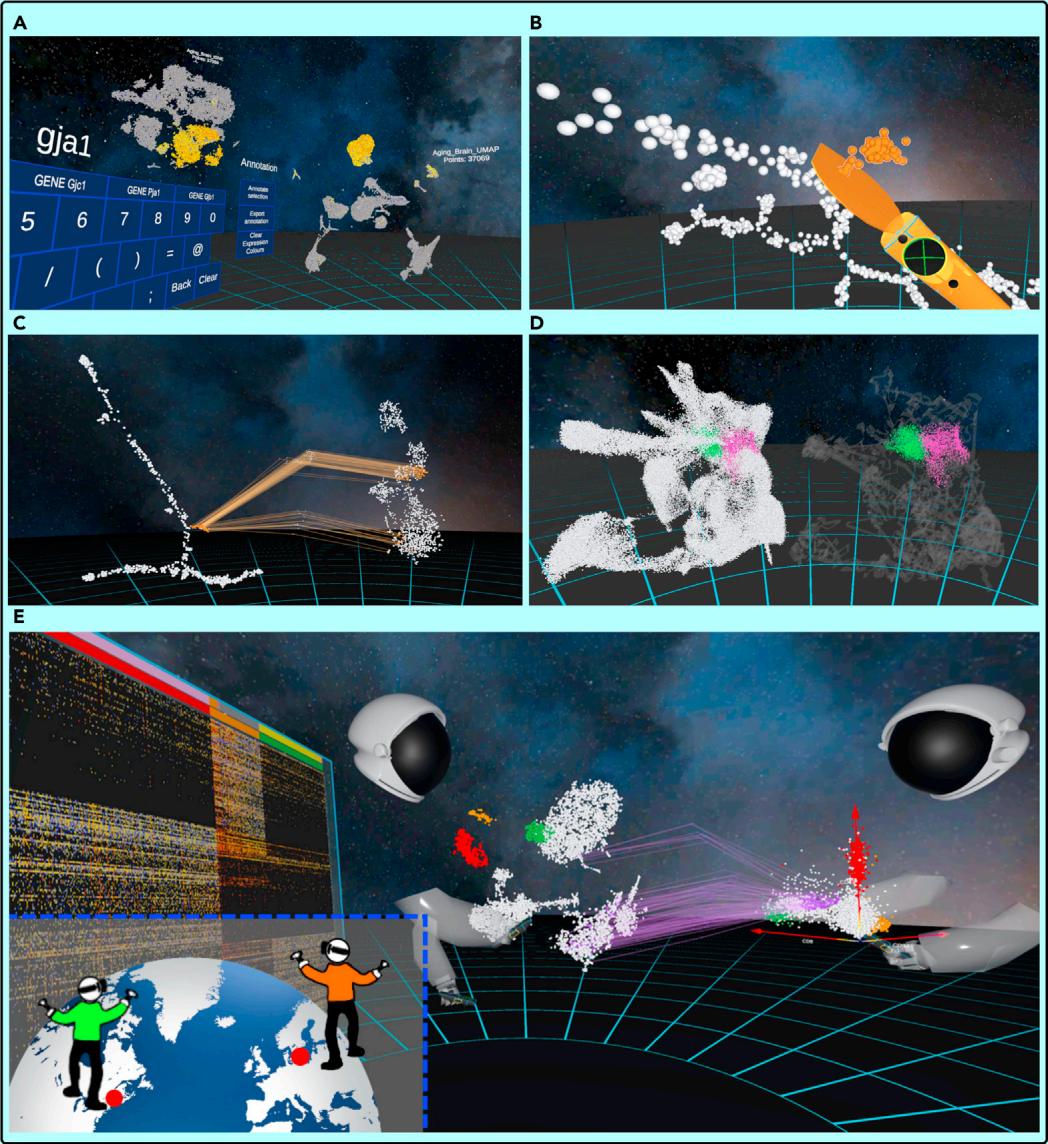
Key features of CellexalVR For a Figure360 author presentation of this figure, see https://doi.org/10.1016/j.isci.2021.103251. (A) A tSNE and UMAP of cells from the aging brain colored by the expression of *Gja1*. (B) Cells can be freehand captured by passing them through the selection tool and clicking the touchpad changes color/initiates a new group. (C) Cells selected from the DDRTree plot (left) are tracked to their counterpart cells in the tSNE plot (right), generating a new graph of points between the two that can also be selected on. (D) Cell types can be colored and spawned in a separate graph if they are difficult to see in dense DR plots. Here, two-types of mesoderm are rendered within a skeleton of the data (right). (E) Multiuser mode showing two users in the same CellexalVR session analyzing the same data together.

At a minimum CellexalVR should be provided with gene expression values and at least one set of DR coordinates (2D or 3D). CellexalVR will also import cell surface marker intensities captured during index sorting/CITEseq and categorical metadata for cells and genes. Figures 3A–3E shows a small selection of features currently available. DR plots can be colored according to gene expression selected using a keyboard in the virtual environment, here the expression of *Gja1* in a tSNE and UMAP of 37k cells from aging mouse brain (Ximerakis et al., 2019) (Figure 3A, Video S1). A key feature of CellexalVR is that cells can be freehand captured into groups using the selection tool where they are colored as they pass through (Figure 3B). Each cell is individually selectable, and to make this possible we engineered a completely new collision detection system to accommodate a large number of points (see STAR Methods). Selecting a new color using the touch-pad initiates a new group of cells, and when two or more groups have been defined the user can (among other things) calculate and display a heatmap of differentially expressed genes (Figure 3E left), generate networks of transcription factors (Figure S3), and also annotate the groups manually with the VR keyboard and export them to a text file. Cells captured in one DR plot can also be traced to their counterparts in other DR plots to visually assess the differences between them (Figures 3C). Here, cells captured from a DDRTree of mouse hematopoietic stem and progenitor cells split into two groups in a tSNE made from the same cells. Furthermore, using the selection tool the cells forming the split can be captured using the centrally generated graph to then determine which genes are differentially expressed between the two (Video S2). Cells can also be colored by user-provided attributes, and for dense maps they can be rendered in a separate plot embedded within a translucent skeleton of the data to orientate their position within the total graph (Figure 3D, Video S3). Here, the two classes of mesoderm from 116k cells assayed during mouse gastrulation shown in Figure 2 are shown where the interface between these two cell-types is much clearer to see when spawned separately.

Video S1. Coloring cells by gene expression, related to Figure 3A Genes are searched for by using the virtual keyboard that queries an SQL database of genes and their expression values. When confirmed all DR plots in the environment are colored according to expression. Heatmap expression colors can be changed using the desktop configuration menu.

Video S2. Making a cell selection, related to Figure 3B The selection tool is activated and the required cells are captured by passing them through the paddle. The cells can be traced to their counterparts in another DR plot which has been calculated using a different method. The selection tool can also be used on the connecting edges to define N groups from which differentially expressed genes can be calculated and displayed in a heatmap which is generated in the session.

Video S3. Coloring cells by attributes, related to figure 3D Two cell types have been selected, and for dense DR plots where the interface between selected types is “buried” the user has the option to render just these groups in a skeleton of the total data to show their relative position.

CellexalVR has been built using video game technology, and we have leveraged this to develop multiuser mode where several individuals can meet in the same CellexalVR session and collaboratively analyze the same data together regardless of geographical location, a significant advantage given many datasets are generated collaboratively over different sites. All actions are synchronized between participants, so each individual will see/experience what their colleagues are doing and vice versa. Figure 3E shows two users analysing data from cord blood mononuclear cells from a CITEseq experiment (Stoeckius et al., 2017) where the right-most user has chosen three surface markers to plot against each other, and has tracked a selection of these cells to the tSNE of the RNA data. The left user has selected groups from the UMAP and created a heatmap of differentials. Each participant will need access to a VR headset, controllers, workstation, and a copy of the input data files. It is also possible for users to join a session using just a desktop computer by navigating the session using a keyboard and mouse. These participants cannot be seen by the VR active users or interact with the session objects, hence we call this “spectator mode”. All voice is transmitted using a third-party application of the user’s choice (Skype/Discord/Zoom to name a few).

We have also developed a more informative way to visualize output from RNA velocity (La Manno et al., 2018) analysis. The velocity of each cell is shown as an animated process where an arrow/point travels the length of the calculated trajectory in a fixed time. The arrows are spawned randomly to avoid overcrowding, and the overall effect shows a “flow” of cells as they traverse through their trajectories. This removes the need to average velocities over a grid for plotting on a 2D map where subtleties can be lost depending on the grid size chosen. These trajectories can also be colored by cell attribute/gene expression adding a further dimension to the information that can be simultaneously visualized. This feature is demonstrated using 142K cells from human fetal liver (Popescu et al., 2019) where we show the trajectories being colored by gene expression level and cluster membership (Video S4). CellexalVR will also pseudotime order cells using simple hand gestures. Using the selection tool, the user traces a path through the data which uses this information to define the start and stop clusters for a slingshot analysis (Street et al., 2018). Returned are a heatmap of genes correlated to the inferred pseudotime. Our implementation allows the user to intuitively define and calculate cell trajectories in 3D reductions on-the-fly. CellexalVR has many other features, and video guides and documentation are provided on the project website.

Video S4. Visualizing RNA velocity in CellexalVR, related to Figure 1C Each cell emits an arrow that travels the length of the calculated vector. The longer the trajectory the redder the arrow. Arrows can be changed to small particles for easier viewing on dense plots, and both emission types can be colored by gene expression or cell attribute. Data from the Liver Cell Atlas reference in the main text.

User comfort has been a key priority during the development of CellexalVR which has been designed to eliminate VR induced motion sickness which is a known issue. The default environment is relatively dark which is easy on the eyes and provides high contrast against the cells. Other environments are available from the configuration menu which also allows the setup of a custom color palette, and change the size and rendering quality of the cells. The dedicated VR headset and controllers provide a smooth, high resolution view of the environment with a wide field of view which is fully tactile and fast. Importantly, CellexalVR is comfortable to use for extended periods of time (1 h +) which is essential for the thorough analysis of complex data. CellexalVR can also export heatmaps, gene networks, cell annotations, screenshots (via the camera tool), and results generated during a session leading to productive and documented sessions. Videos can also be created and exported using the “fly-by” feature. The user charts a course around the objects they wish to capture over which a virtual camera will pan and record the scene. The captured images are then automatically compiled into an mp4 producing a smooth high-resolution movie. For those not familiar with VR we have created an interactive training session where the user is familiarized with the environment and shown how to perform basic functions in CellexalVR.

### Comparison to other software

There are choices of GUI based tools aimed at enabling all researchers’ to visualize single-cell data which have been comprehensively compared. Most of these tools are catered toward the navigation of 2D reductions which we have already shown to be sub-optimal for complex datasets, but moreover the interface layouts are generally rigid. Where tools allow the display of several reductions, they can usually only be shown one at a time. The same also applies to those tools that allow 3D reductions to be displayed; therefore, easy cross-comparison is not possible which is particularly essential in a multi-omics setting. CellexalVR with its open VR environment allows an intuitive build-up of in session objects as they are generated which can be arranged or removed as the user wishes. CellexalVR will also calculate differentially expressed and pseudotime trajectories from cells selected in-session whereas other tools require pre-calculation, or are limited to two-group comparisons. In all cases there is a lack of true multi-user mode where several researchers can collaboratively analyze data in real-time and in the same location.

There are currently three other projects in preprint that allow users to visualize single-cell data in stereoscopic 3D, starmapvr (Yang et al., 2018), singlecellvr (Stein et al., 2020), and Theia (Bressan et al., 2021) (which will also handle spatial data). Both starmapvr and single-cellvr utilize Google cardboard where a mobile phone is placed inside a visor to form a rudimentary 3D viewer. There is no connection to a computer for high-resolution GPU accelerated performance and in-session calculations. Moreover the user’s hands are not present in the VR environment to directly manipulate the data or activate functions, rather relying on gaze control and/or the keyboard which is significantly less intuitive and far slower to use.

CellexalVR uses high-resolution *true* VR equipment where all user motions are smoothly tracked (6 degrees of freedom vs 3 for cardboard) for optimum comfort and full hands-on interaction with objects in the environment. A table comparing features between CellexalVR and other multi-function GUI based tools is shown in Table S1.

#### Preparing data for CellexalVR

CellexalVR requires several input files which are detailed on the project website, but to make it easy we have created an R package (cellexalvrR) to help prepare them. Those using Seurat and AnnData objects can use our “as_cellexalvrR” function which will produce a cellexalvrR object that is exported using “export2cellexalvr”. To enable RNA velocity visualization users should supply coordinates denoting the destination of each cell alongside the coordinates of the DR method used. All details and tutorials regarding use of the R package can be found at https://cellexalvr.med.lu.se/cellexalvrr-vignette. Importing data from multiple analysis platforms is a key challenge and one that we will continue to work on.

#### Data types handled by CellexalVR

The data supplied to CellexalVR is flexible regarding experimental sources. The expression matrix and DR coordinates used as input can be derived from other assays. For example, gene activities from single-cell ATACseq (Figure S4), and we have also used CellexalVR to visualise CyTOF and FACS data. In the near future we will routinely see multi-modal data from the same cell (such as RNA and epigenetic information), and CellexalVR will be the perfect environment to unify all visualizations from these complex and information rich experiments. There is also a clear application of CellexalVR to spatial transcriptomics experiments which will form a future update.

## Discussion

The analysis and interpretation of scRNAseq experiments are heavily reliant on visualization to draw correct conclusions, and how many dimensions to reduce single-cell data to is an important consideration. The limitless space available in virtual reality (VR) allowed us to rethink how 3D data objects are displayed, interacted with, and connected together for a better integrated view of single-cell experiments. We have demonstrated that virtual reality is an excellent medium in which to fully scrutinize this complex data in a manner which is intuitive and fully collaborative. VR technology will continue to evolve, as will CellexalVR as we improve current functionality and develop new ones to keep apace of single-cell and other higher-dimensional methodologies.

### Limitations of the study

As single-cell datasets become bigger with respect to the number of cells, the computational burden increases accordingly. CellexalVR can visualize a very large number of cells (>500k) but backend calculations such as identifying differentially expressed genes will take longer to complete depending on how many cells/groups are selected. We are continuously working on CellexalVR to improve speed, and methods will update as they become available.

## STAR★Methods

### Key resources table

**Table:** 

REAGENT or RESOURCE	SOURCE	IDENTIFIER
**Software and algorithms**		

Unity 2019.1.8f1	Unity Technologies	https://unity.com/
SteamVR	Valve	https://store.steampowered.com
Photon Unity Networking 1.92	Photon Engine	https://www.photonengine.com/pun
VRTK 3.3	Extend Reality Ltd	https://vrtoolkit.readme.io/
Octree	Meagher (1982)	N/A
Quickhull	Barber et al. (1996)	N/A
Intersecting polygons	O'Rourke et al. (1982)	N/A
R 4.0.0	R Foundation	https://cran.r-project.org/bin/windows/base/old/4.0.0/
CellexalVR	This paper	https://doi.org/10.5281/zenodo.5513575
cellexalvrR	This paper	https://doi.org/10.5281/zenodo.5513687

**Deposited data**		

Ageing brain	Ximerakis et al. (2019)	GEO: GSE129788
Mouse HSPC	Nestorowa et al. (2016)	GEO: GSE81682
CITEseq	Stoeckius et al. (2017)	GEO: GSE100866
Mouse Gastrulation	Pijuan-Sala et al. (2019)	GEO GSE87038
Liver Cell Atlas	Popescu et al. (2019)	GEO: GSE127980

### Resource availability

#### Lead contact

Further information and requests for resources should be directed to and will be fulfilled by the lead contact, Shamit Soneji (shamit.soneji@med.lu.se).

#### Materials availability

This work did not generate any materials.

### Method details

The CellexalVR executable is available from the project website at https://cellexalvr.med.lu.se/download along with extensive documentation and video tutorials at https://www.cellexalvr.med.lu.se/explore. The data used here is also available from the project website and cited references.

The source code for the CellexalVR is available at https://github.com/sonejilab/cellexalvr which is coded in C#, and the accompanying R package cellexalvrR can be installed directly from GitHub https://github.com/sonejilab/cellexalvrR. For those wanting to modify CellexalVR, we have a developers guide at https://cellexalvr.med.lu.se/programmers-guide.

#### Software requirements

• CellexalVR executable (https://www.cellexalvr.med.lu.se/download)
• R 4.0 or greater.
• Rtools (https://cran.r-project.org/bin/windows/Rtools/) which is used to compile sub-routines in cellexalvrR.
• Pandoc (https://pandoc.org/installing.html) which is used to compile HTML reports.
• cellexalvrR (https://github.com/sonejilab/cellexalvrR).
• SteamVR (which should be installed automatically when the headset is setup).

#### Hardware requirements

Users require a VR ready workstation/laptop with a suitable graphics card (Steam recommends an NVIDIA GTX1060 or higher) running Windows 10. CellexalVR will work with any SteamVR compatible headset. The HTC Vive and Valve Index were used during development. Compatibility for other VR systems will be added. These VR systems are readily available, and are priced for the home consumer.

CellexalVR was developed on a gaming class workstation comprising an Intel i7 processor, 16GB RAM and an NVIDIA GTX1080 graphics card.

#### CellexalVR

CellexalVR is comprised of two main components. The VR user interface and a backend R package.

#### CellexalVR virtual reality user interface

The VR user interface is built using Unity Engine (https://unity.com/) that handles tasks such as rendering the frames that are displayed on the computer's monitor and in the user's headset, collecting input from the keyboard and the controllers and forwarding events that trigger certain actions and handling all physics simulation. Unity comes with an editor which is the primary development environment CellexalVR was created with. CellexalVR uses several Unity assets that are libraries containing scripts that handle parts of the program logic. SteamVR (https://steamcommunity.com/steamvr) and OpenVR (https://github.com/ValveSoftware/openvr) handle communication between the computer, headset and controllers, and VRTK (https://github.com/thestonefox/VRTK) handles basic interaction logic such as the grabbing of objects.

#### cellexalvrR

Input files for CellexalVR are produced in R using our cellexalvrR package (https://github.com/sonejilab/cellexalvrR). A guide is provided at https://www.cellexalvr.med.lu.se/cellexalvrr-vignette where we give working examples showing how to make a CellexalVR object from Seurat/AnnData/scvelo processed data and export it to files CellexalVR will accept. We also give an example of how to make and export RNA velocity embedding in 3D for visualisation. A detailed description of the file formats can be seen at https://cellexalvr.med.lu.se/manual_introduction. During export the following files are produced:

• **An R object containing all data.** This is loaded into an R server session when the data is loaded in CellexalVR in preparation for in-session calculations.
• **An SQLite database** that Unity uses to draw expression values from when colouring cells by gene expression and rendering heatmaps. SQLite is used because it carries significant speed advantages.
• **Basic text files** that are used to populate the environment (DR plots and metadata) when data is loaded.

In-session calculations are also performed by cellexalvrR. When a data set is loaded an R server is created and initialised with the expression data. Differentially expressed genes for heatmaps are identified using the Wilcox test (implemented in C++ for speed) and clustering is performed using hierarchical clustering for the top *N* marker genes (default is 250). Transcription factor (TF) networks within selected groups of cells can be calculated using the rho value (default) from the propr package, or partial correlations from the ppcor package. The top 130 interactions are returned by default, but is user configurable. All heatmaps and networks are rendered in the CellexalVR UI. TFs are defined as those in the AnimalTFDB database (http://bioinfo.life.hust.edu.cn/AnimalTFDB/).

Pseudotime trajectories are calculated by partitioning the selected cells into four clusters which are used for a slingshot analysis. Cells are ordered according to the returned pseudotime and split into ten even sized colour groups, shown in the returned heatmap of genes that are correlated to pseudotime.

At a minimum cellexalvrR should be provided with:

• ***A matrix of gene expression data (C cells (col) x G genes (row))*.** This is processed by cellexalvrR to an SQLite database that CellexalVR queries when needed.
• ***At least one set of DR coordinates placing the cells in 3D space (C cells x 3)*.** This can be from any DR methods the user deems suitable and CellexalVR will accept more than one DR table. These are exported as 4-column text files (∗.mds) with the Cell ID in the first column. If RNA Velocity trajectories have been added these will be 7-column.mds files.

In addition to these, further optional files can be imported. These are:

• ***Surface marker intensities (C cells x S surface markers)*.** These are recorded when cells have been index sorted. If using CITEseq, these expression values go into this table.
• ***Cell type information (C cells x T types)*.** These allow the user to label each cell as being of a certain type, which can then be displayed in the CellexalVR session. Cells are marked as belonging to a class with a “1”, or “0” otherwise.
• ***Metadata for cells (C cells x M meta)*.** Further labels for cells, for example cell-cycle stage.
• ***Metadata for genes (G genes x M meta)*.** For example, marking genes if they belong to a particular category such as transcription factors, epigenetic factors, or code surface proteins.

More details on how to create and export CellexalVR ready files can be seen at https://www.cellexalvr.med.lu.se/cellexalvrr-vignette.

**Output from CellexalVR**. During a CellexalVR session the user has the option to document what they have done using various tools and methods:

• Capture images using the “Camera tool” button placed on the menu controller.
• Save text files of annotated Cell IDs when the user has used annotation mode.
• Images of heatmaps can be saved by clicking on the “Save” button to the left of the heatmap.
• Images of TF networks can be saved by clicking on the “Save as image” button on the surrounding border.
• An adjacency list of a network can be saved by clicking on the “Save as text file” button on the surrounding border.
• The modified R object is returned containing all the user captured groups.
• An html report is generated when the user clicks the “Save session” button on the menu, or when the program is exited. Everything the user has decided to save during a session is compiled into an HTML report with figures (heatmaps and networks) and associated p-values and FDR's for the differentially expressed genes.

Compartmentalising CellexalVR this way means bioinformaticians can alter the R package to customise the underlying methods without needing knowledge of C# (the language used by Unity). The files made by cellexalvrR include an R object used for backend calculations, the dimension reduced data coordinates, metadata, surface marker intensities (if present), and a SQLite database which the VR environment accesses to retrieve expression values for display.

cellexalvrR can be installed directly from github using the command:

devtools::install_github("sonejilab/cellexalvrr")

#### RNA velocity

Video S4 was made by aligning reads from the Liver Cell Atlas using kallisto-bustools (Melsted et al., 2021) using loompy's “fromfq” function (http://loompy.org/). The resulting loom file was processed through scvelo (Bergen et al., 2020) to calculate the RNA velocities and the output files were created using cellexalvrR. Trajectories were based on two 3D embeddings, PHATE (Moon et al., 2019) and UMAP.

#### Collision detection

Selection in Unity is made possible using “colliders” that are placed in each point that detects collision with the selection tool. The native implementation in Unity is inefficient and would cause lag for more than 30k cells. To overcome this, a new collision detection system using Octrees (Meagher, 1982) and raycasts was engineered. The visual form of the octree is used in sub-graphing to show a skeleton of the data (as seen in Figure 3D) This is described at https://www.cellexalvr.med.lu.se/programmers-guide along with a detailed overview of the code structure and how to navigate it.

#### Multi-user mode

Multi-user mode is facilitated via the Photon Unity Networking (PUN, https://www.photonengine.com/en-US/PUN) that works by sending packages that contain information about events between the users through Remote Procedure Calls (RPCs). The RPCs ensure that each user's session is synchronised with all others. The data files are *not* transmitted over RPCs, therefore each user must have a copy of the data being analysed locally on the workstation they are using. The maximum number of participants during a multi-user session is 16, and spectator mode does not require gaming class hardware to run.

Head models were downloaded from NASA (https://nasa3d.arc.nasa.gov) and the arm models were made in Blender (https://www.blender.org/).

#### Cluster/cell-type overlap analysis

Controlled user studies have shown that, compared to 2D, 3D projections improves the ability to discriminate datapoint clusters visually (Poco et al., 2011). Furthermore, it has been shown that visual inspection of datapoints is better when 3D embeddings are explored interactively rather than as fixed axis animated movies (Sanftmann and Weiskopf, 2012).

Two different approaches were taken to measure the degree of overlap/separation between cell-types and clusters in 3D and 2D projections of scRNAseq data, 1) an entropy based method, and 2) measuring area/volume overlap between convex hulls. For example, two cell populations that seemingly overlap in a 2D projection can be resolved into separate populations when the cells are projected onto three dimensions instead, and this can be seen when rotating the 3D plot. To mimic this, for both the entropy and convex hull method we computationally rotate the 3D projections of the data and then “flatten” the cells to a 2D representation before calculating pairwise the overlap between cell type/clusters. We retain the value corresponding to the least overlap and compare that to the data when projected onto two dimensions only. In both entropy and hull methods we used UMAP and tSNE using three large and well annotated datasets; 116k cells from mouse gastrulation, 100k cells from mouse organogenesis (Cao et al., 2019), and the Mouse Cell Atlas (Han et al., 2018).

**Entropy Method** This approach uses the *Shannon Index* (*SI*) as the measure of overlap. To start, a grid is placed over the UMAP/tSNE dividing it into a number of different sectors. For each sector the *SI* is calculated using the following:

(Equation 1) $$SI=-\sum_{i=1}^{R}p_{i}\;\text{ln}\;p_{i}$$

where *p*_*i*_ is the proportion of cells belonging to the *i*th population, *R* is the number of different populations, since we are doing pairwise comparisons *R*=2. Lastly the *SI* of each sector is summed together. To ensure the placement of the grid is not a major influential factor of the result, we shift the grid stepwise and use the position that gives the lowest overall *SI* value. This is done for both the 2D and 3D setting.

As explained above, the 3D graphs are flattened to 2D after systematically rotating the graph to cover all angles. For each of these projection angles a grid is placed and the *SI* is calculated the same way as in Equation 1. The projection that gives the lowest *SI* value is picked as the best viewing angle and this is the value that is then compared to the 2D graph. If the value is lower for the best viewing angle in the 3D graph it means the third dimension helped separate these populations since you were able to find an angle where the cell populations or clusters had less overlap compared to the 2D projection. Code for this method can be obtained at https://github.com/sonejilab/entropy_2dvs3d.

**Convex Hull Area Method** In a 2D projection of the data, convex hulls were calculated for each defined cell type or cluster. The convex hulls were then compared pairwise and the overlap was calculated in terms of area. The area of the overlap between the two convex hulls was calculated as a percentage of the smallest hull in terms of area. In 3D projections, the populations were first flattened onto 2 dimensions through 2048 different angles in order to emulate what a user sees from a given angle. For each pair of cell type/clusters the viewing angle with the least amount of overlap in terms of area was found using an exhaustive search. A convex hull of the overlap was calculated and populations were compared pairwise as for the 2D setting.

The convex hulls were calculated using the Quickhull algorithm (Barber et al., 1996) and the overlapping area between two given convex hulls were calculated (O'Rourke et al., 1982). Code for this method can be obtained from https://github.com/sonejilab/2d_vs_3d.

## Data Availability

• All data used here is publicly available, and the accession IDs are in the key resources table. • All original code has been deposited at Zenodo and is publicly available as of the date of publication. DOIs are listed in the key resources table. • Any additional information required to run the software reported in this paper is available from the lead contact upon request.

## Supplemental information

Document S1. Figures S1–S4 and Table S1

Figure360. An Author Presentation of Figure 5
