# Supplementary material for: CellexalVR: A virtual reality platform to visualize and analyze single-cell omics data
Source: iScience. 2021 Oct 27;24(11):103251. doi: 10.1016/j.isci.2021.103251 (PMC8609247; doi:10.1016/j.isci.2021.103251)
Supplement: Document S1. Figures S1–S4 and Table S1 [file mmc1.pdf]

iScience, Volume *24*

Supplemental information

CellexalVR: A virtual reality platform to visualize and analyze single-cell omics data

Oscar Legetth, Johan Rodhe, Stefan Lang, Parashar Dhapola, Mattias Wallergård, and Shamit Soneji

Supplementary Figure 1: Boxplots showing comparison of 2D and 3D dimension reduction of scRNAseq data. Related to Figure 2.

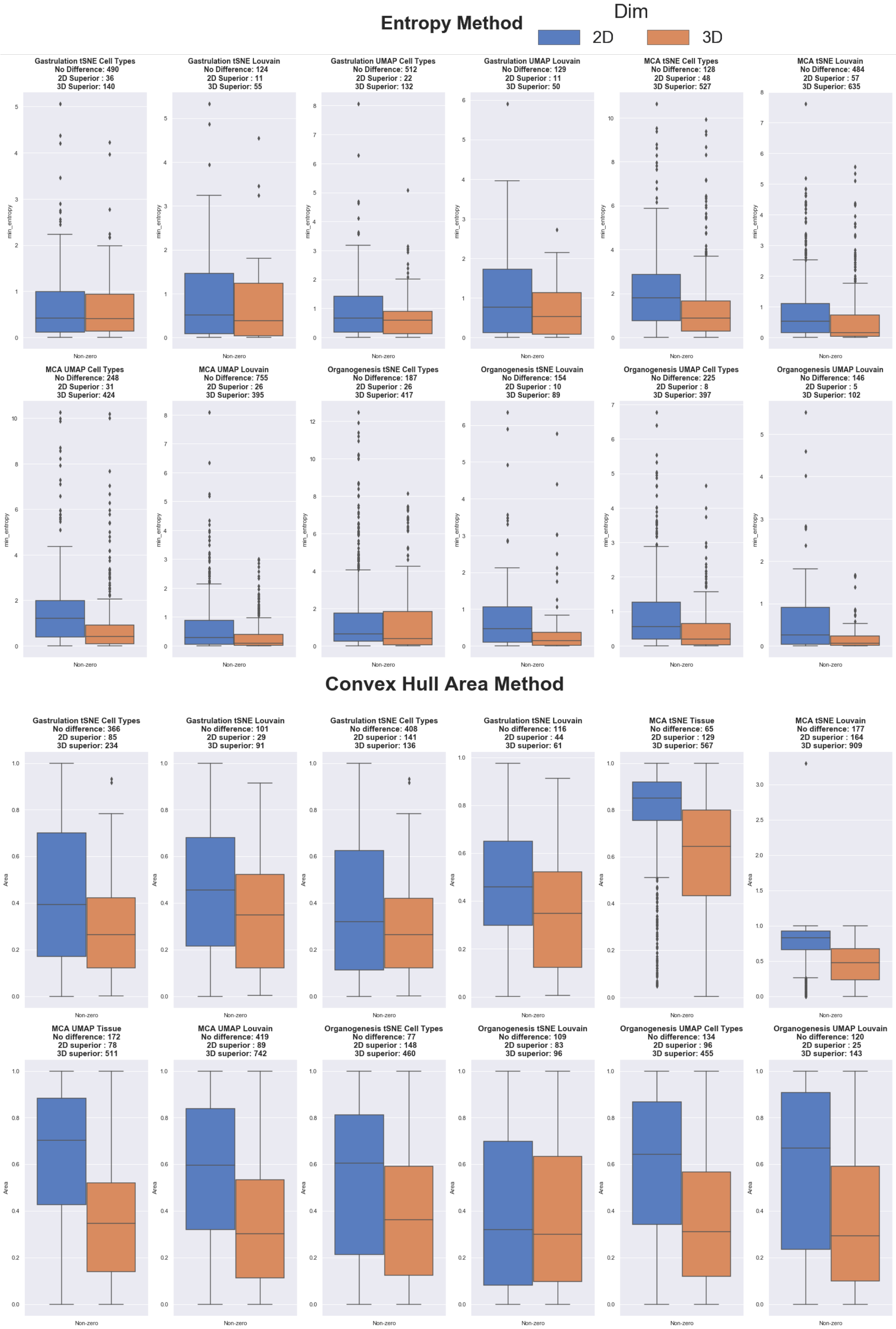

Figure S1: A comparison of 2D (blue boxes) vs 3D (orange boxes) dimension reduction of scRNAseq using three datasets implementing UMAP and tSNE. Boxplots show the distribution of entropies (top 12), or, area overlap of convex hulls (bottom 12) which were calculated comparing pairwise the overlap between cell types and between Louvain clusters. Overall we observe projecting the cells onto 3 dimensional data will allow close but distinct populations to be visually resolved to a greater extent when compared to 2D projections. Boxplots show the median (mid-line), Q1 and Q3 (box), and whiskers to 1.5x the IQR.

Supplementary Figure 2: Heatmaps showing comparison of 2D and 3D dimension reduction of scRNAseq data. Related to Figure 2.

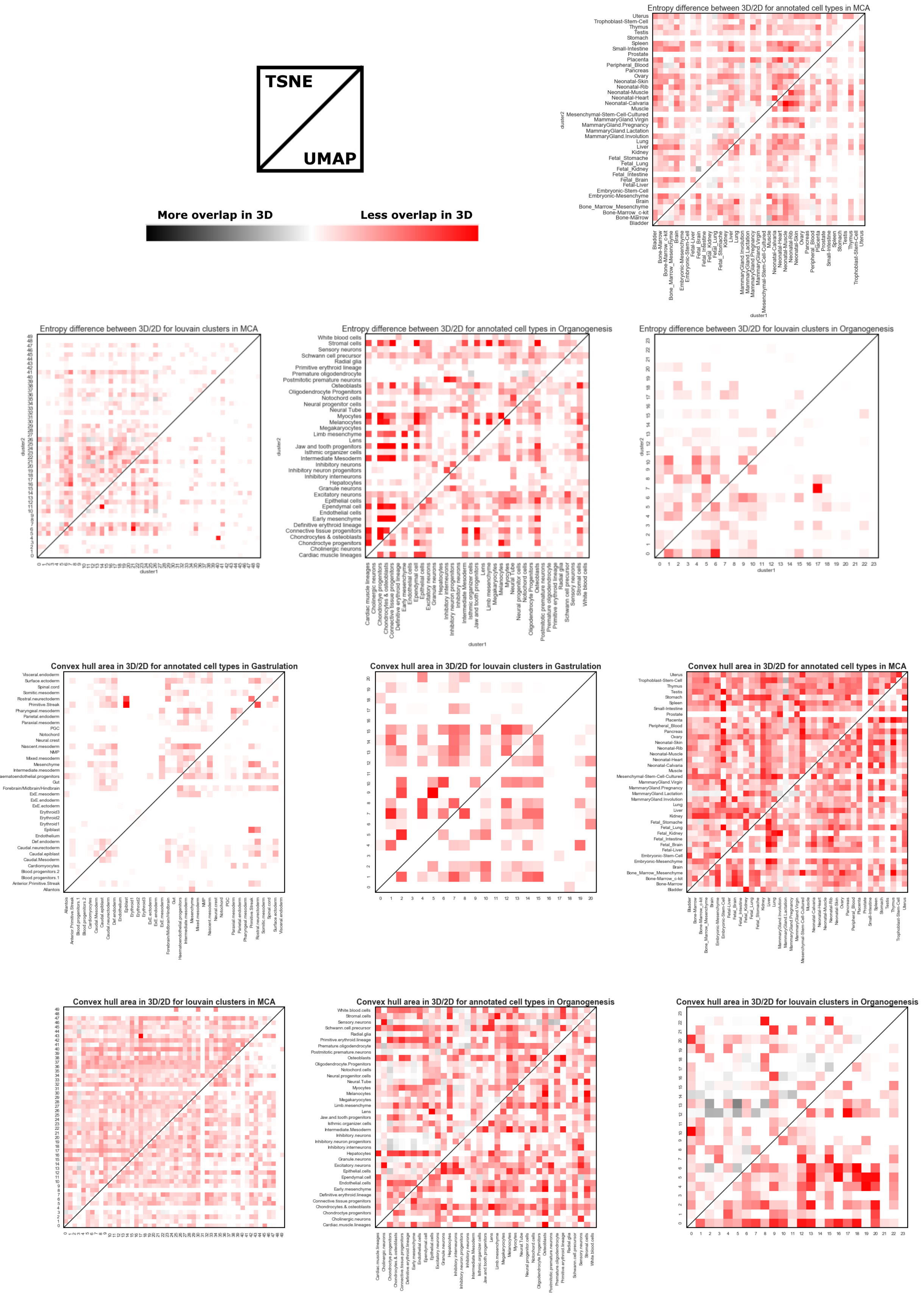

Figure S2: Heatmaps of the data shown in FigS1. Each shows the pairwise difference in entropy (top four), or, area overlap of convex hulls (bottom 6) which were calculated comparing the overlap between cell types and between Louvain clusters. Red denotes less cell mixing in 3D reduced data which is the dominant trend within these datasets regardless of DR method used.

Supplementary Figure S3: Comparing TF-TF networks from selected groups of cells

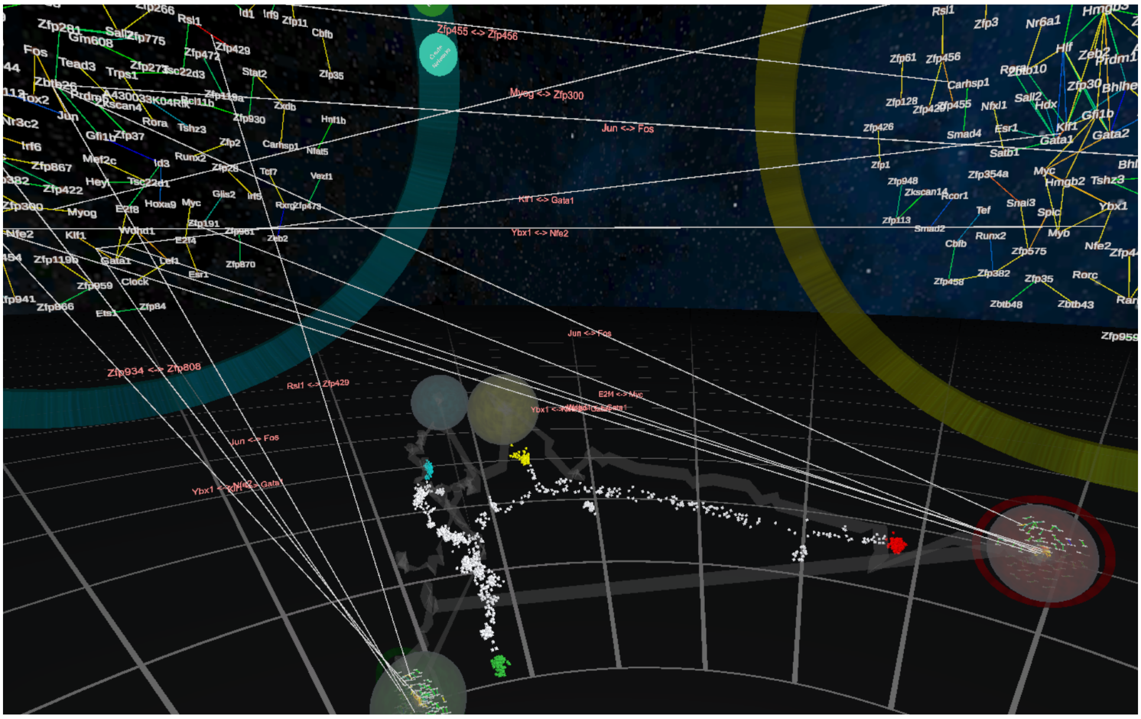

Figure S3: TF-TF correlation networks that have been calculated for each of the groups captured in the dimension reduced plot in the background. Each network is rendered in a sphere that is embedded in a abstract representation of the DR graph to show each networks originating group. Here, two networks have been clicked and expanded, and common TF-TF pairs have been toggled and highlighted to show where commonalities lie between groups. Related to STAR methods.

Supplementary Figure S4: Colouring cells by gene expression

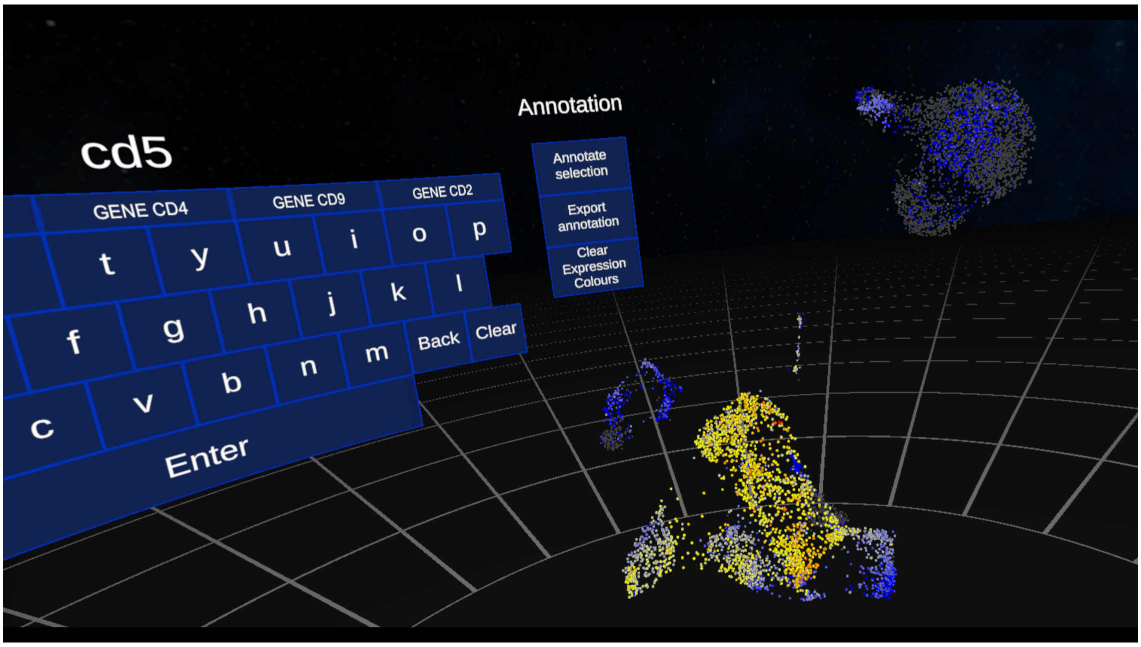

Figure S4: A UMAP generated from single-cell ATACseq from 7866 PBMC cells. Here, each cell is coloured by the activity of cd5. Data was processed according to the Seurat documentation `https://satijalab.org/seurat/v3.2/atacseq_integration_vignette.html` and exported using cellexalvrR. Related to STAR methods.

Supplementary Table 1. A comparison of visualisation tools. Related to STAR Methods

	CellexalVR	starmapVR	singlecellVR	Cerebro	cellxgene	iSEE	Loom.viewer	scSVA	SCope	Single.Cell.Explorer	UCSC.Cell.Browser	ASAP
Interactivity	✔	-	-	✔	✔	✔	-	✔	✔	✔	✔	✔
Cloud support	-	-	-	-	✔	-	-	✔	✔	-	-	-
SaaS	-	✔	✔	-	✔	-	-	-	-	✔	✔	✔
VR	6DoF	3DoF	3DoF	-	-	-	-	-	-	-	-	-
Ease of cell selection	✔	-	-	✔	✔	✔	-	✔	✔	-	✔	✔
View 3D embeddings	✔	✔	✔	✔	-	-	-	✔	-	-	-	✔
Select *within* 3D embeddings	✔	-	-	-	-	-	-	-	-	-	-	-
Zoom in/out	✔	✔	✔	✔	✔	✔	-	✔	✔	-	✔	✔
Multiple embeddings	✔	-	-	✔	✔	✔	✔	-	✔	-	✔	✔
Highlight gene expression	✔	✔	✔	✔	✔	✔	-	✔	✔	-	✔	✔
Highlight metadata	✔	✔	✔	✔	✔	✔	✔	✔	-	✔	✔	✔
Differential expression	✔	-	-	✔[3]	✔	✔	-	-	-	-	-	✔
Manual cell annotation	✔	-	-	-	✔	-	-	✔	✔	-	-	-
Cell-type prediction	-	-	-	-	-	-	-	-	-	✔	-	
Web page loads fast	NA	✔	✔[4]	NA	✔	-	✔	✔	✔	✔	✔	✔
Comparable DR plots in session[1]	✔	-	-	-	-	-	-	-	-	-	-	-
Simultaneous multi-omics	✔	-	-	-	-	-	-	-	-	-	-	-
Pseudotime	✔*	-	✔	✔	✔	-	-	-	-	-	-	-
RNA velocity	✔	-	✔	-	-	-	-	-	-	-	-	-
Session reports	✔	-	-	-	-	-	-	-	-	-	-	-
Real-time collaboration mode	✔	-	-	-	-	-	-	-	-	-	-	-
Spatial transcriptomics[2]	-	✔	-	-	-	-	-	-	-	-	-	-

Table 1: A comparison of CellexalVR to other single-cell visualisation tools based on a previous evaluation (Çakir et al, reference in main text). DoF refers to the degrees-of-freedom regarding body tracking provided by a VR equipment. 3DoF is head rotation only (Google cardboard), where 6DoF provides full body tracking including the users hands in the environment and body position within the room. CellexalVR uses 6DoF systems. *CellexalVR allows computation in session using slingshot, others require it to be pre-computed. [1] Multiple embeddings can be viewed simultaneously and be directly cross compared on a per cell basis. [2] Most tools can handle spatial transcriptomics data as it is handled as single-cell data, but we only count it if the software can handle source images.[3] Cerebro will calculate differentials prior to visualisation using Seurat, and not on selections made in-session.[4] Slows as datasets get bigger. All of these projects are under active development with starmapvr, singlecellvr, cellxgene, scSVA in preprint at the time of writing.
